# A rice dual-localized pentatricopeptide repeat protein is involved in organellar RNA editing together with OsMORFs

**DOI:** 10.1093/jxb/ery108

**Published:** 2018-03-17

**Authors:** Haijun Xiao, Yanghong Xu, Chenzi Ni, Qiannan Zhang, Feiya Zhong, Jishuai Huang, Wei Liu, Leilei Peng, Yingguo Zhu, Jun Hu

**Affiliations:** State Key Laboratory of Hybrid Rice; Engineering Research Center for Plant Biotechnology and Germplasm Utilization of Ministry of Education, College of Life Sciences, Wuhan University, Wuhan, China

**Keywords:** Chloroplast, dual-localized, mitochondrial, OsMORFs, PPR, RNA editing

## Abstract

In flowering plants, various RNA editing events occur in the mitochondria and chloroplasts as part of post-transcriptional processes. Although several pentatricopeptide repeat (PPR) proteins and multiple organellar RNA editing factors (MORFs) have been identified as RNA editing factors, the underlying mechanism of PPRs and the cooperation among these proteins are still obscure. Here, we identified a rice dual-localized PPR protein, OsPGL1. The loss of function of *OsPGL1* resulted in defects in both chloroplast RNA editing of *ndhD*-878 and mitochondrial RNA editing of *ccmFc*-543, both of which could be restored in transgenic complementation lines. Despite synonymous editing of *ccmFc*-543, the loss of editing of *ndhD*-878 caused a failed conversion of serine to leucine, leading to chloroplast dysfunction and defects in the photosynthetic complex; the results of additional experiments demonstrated that OsPGL1 directly binds to both transcripts. Interactions between three OsMORFs (OsMORF2/8/9) and OsPGL1 both *in vitro* and *in vivo* were confirmed, implying that OsPGL1 functions in RNA editing via an editosome. These findings also suggested that OsMORFs assist with and contribute to a flexible PPR–RNA recognition model during RNA editing. These results indicate that, in cooperation with PPRs, OsPGL1 is required for RNA editing. In addition, our study provides new insights into the relationship between RNA editing and plant development.

## Teaser

A dual-localized PPR protein, OsPGL1, is required for simultaneous RNA editing in mitochondria and chloroplasts. OsPGL1 binds to two distinct target transcripts directly and cooperates with OsMORFs.

## Introduction

RNA editing is broadly defined as a post-transcriptional process whereby the sequence of an RNA molecule is altered from that of its master DNA (Covello and Gray, 1989). RNA editing is widespread among eukaryotic cells and is particularly abundant in plant mitochondria and chloroplasts. In contrast, RNA editing rarely occurs in animals; only a few cases have been reported, including C-to-U RNA editing catalyzed by the apolipoprotein B mRNA editing enzyme (APOBEC) (Teng *et al.*, 1993), U-to-C RNA editing by a putative editing reaction reported in mouse mitochondria (Villegas *et al.*, 2002), and adenosine (A)-to-inosine (I) editing by adenosine deaminase acting on RNA (ADAR) (Kim *et al.*, 1994; Mehta and Driscoll, 2002; Melcher *et al.*, 1996). In plants, RNA editing also includes C-to-U, U-to-C, and A-to-I conversions (Takenaka *et al.*, 2013*b*). The majority of editing in plants involves mitochondrial and plastid transcripts; however, A-to-I editing of cytosolic tRNAs also occurs (Chateigner-Boutin and Small, 2010). C-to-U RNA editing is the most frequent editing event in plants. More than 40 plastid and more than 600 mitochondrial C-to-U RNA editing sites have been reported in Arabidopsis (Bentolila *et al.*, 2013*b*; Ruwe *et al.*, 2013), while 21 plastid and 491 mitochondrial C-to-U editing sites have been reported in rice (Corneille *et al.*, 2000; Notsu *et al.*, 2002). In humans, APOBEC proteins can act on ssRNA or ssDNA. For example, APOBEC3 proteins can deaminate cytidines to uridines in ssDNA (McDougall *et al.*, 2011). APOBEC3G (APO3G) binds efficiently to both ssDNA and ssRNA but can only deaminate cytidines to uridines in ssDNA (Iwatani *et al.*, 2006). Recently, the fusion of APOBEC together with catalytically dead Cas9 (dCas9) or other Cas9 variants in a clustered regularly interspaced short palindromic repeat (CRISPR) system resulted in the genomic editing of single bases in mammals, yeasts, and plants (Hess *et al.*, 2016; Kim *et al.*, 2017; Lu and Zhu, 2017; Ma *et al.*, 2016; Zong *et al.*, 2017). However, in plants, the deaminase activity still needs to be confirmed and elucidated. Researchers have proposed that RNA editing activity is governed by RNA-binding pentatricopeptide repeat (PPR) proteins, and depending on their similarity to cytidine deaminases that contain the DYW motif, DYW-subclass PPR proteins are considered the main RNA editing factors (Salone *et al.*, 2007).

To date, several non-PPR editing factors, such as RNA editing factor interacting proteins (RIPs)/multiple organellar RNA editing factors (MORFs), organelle RNA recognition motif (ORRM) proteins, organelle zinc-finger (OZ) proteins, and protoporphyrinogen oxidase 1 (PPO1), have been identified as components of the plant RNA apparatus (Sun *et al.*, 2016). Nine MORF proteins exist in Arabidopsis, and seven exist in rice, including two MORF2-like (Os06g02600, Os04g51280), one MORF3-like (Os03g38490), one MORF1-like (Os11g11020), one MORF9-like (Os08g04450), and two MORF8-like (Os09g04670, Os09g33480). MORF8/RIP1, a protein dually targeted to plastids and mitochondria, interacts with RARE1, a PPR protein required for RNA editing in Arabidopsis chloroplasts (Bentolila *et al.*, 2012). MORF2 and MORF9 are both targeted exclusively to plastids and can affect the majority of RNA editing sites in Arabidopsis chloroplasts (Takenaka *et al.*, 2012). MORF2/8/9 can interact with ORRM6, which is involved in the RNA editing of *psbF*-C77 and *accD*-C794 in Arabidopsis (Hackett *et al.*, 2017). Furthermore, MORFs can also interact with each other to form a heterodimeric or homodimeric complex, suggesting that a more complicated regulatory mechanism is at play in plants (Takenaka *et al.*, 2012).

The PPR protein family is characterized by degenerate motifs consisting of 35 amino acids arranged as tandem repeats of 2–25 such elements in a pair of antiparallel double alpha-helices (helices A and B) (Small and Peeters, 2000; Yin *et al.*, 2013). PPR proteins are encoded by the nuclear genome in eukaryotes and are more abundant in plants; more than 400 members of this group exist in land plants (Cheng *et al.*, 2016). PPR proteins can be divided into two subfamilies, P and PLS. P subfamily proteins contain tandem arrays of canonical 35 amino acid (P) PPR repeats, whereas the PLS subfamily proteins are characterized by triplets of P, L (i.e. long—35 to 36 amino acids in length), and S (i.e. short—31 amino acids in length) motifs (Lurin *et al.*, 2004). PPR proteins are involved in many post-transcriptional processes in chloroplasts and mitochondria, including RNA editing, splicing, and cleavage, as well as RNA stability and translation (Schmitz-Linneweber and Small, 2008). Most PPR proteins are targeted to either mitochondria or chloroplasts, but some present dual localization. Exclusively, MPR25/AEF1 has been isolated as a possible editing factor for both organelles. However, it is still unclear whether this protein is indeed dual-localized in both organelles (Yap *et al.*, 2015).

Here, we studied a novel dual-localized PPR gene, *OsPGL1* (*Os12g06650*), in rice; this gene is required for the RNA editing of two different *cis* elements. The loss of editing of *ndhD*-878 caused the failure of conversion of serine to leucine, while the loss of editing of *ccmFc*-543 did not cause a change in valine, both of which are extremely conserved amino acids in plants. The loss of function of *OsPGL1* caused a pale green leaf phenotype, resulting from a defective photosynthetic complex during chloroplast development. Further investigation showed that OsPGL1 functions together with OsMORF2/8/9 and, via its 10 PPR motifs, directly binds to *ndhD* and *ccmFc*. These results confirmed that PPR proteins and OsMORFs are required for RNA editing and that both types of proteins function together via a complex editosome.

## Materials and methods

### Plasmid construction and transformation

Two target sites 20 bp upstream of the protospacer-adjacent motif sequence (PAM) in accordance with the recognition principle of the CRISPR/Cas9 system were designed for *Os12g06650*, after which the specificity was analyzed by CAS-OFFinder (http://www.rgenome.net/cas-offinder) (Supplementary Table S1 at *JXB* online). The target sequence joint was linked to the gRNA-U3 and gRNA-U6 vectors, after which two rounds of nested PCR were performed. The PCR products were subsequently linked to a CRISPR/Cas9 vector. The construction was verified via PCR and sequencing. Calli derived from *Oryza sativa* L. *japonica* Zhonghua 11 (ZH11) were used for *Agrobacterium*-mediated transformation. Wild-type (WT) and CRISPR/Cas9 knockout lines were grown in a paddy field and greenhouse in Wuhan, China, under appropriate management.

### Scanning electron microscopy and transmission electron microscopy assays

For SEM assays, samples were prepared as described previously (Zhou *et al.*, 2011). Young rice leaves were cut with a razor into small sections and immediately immersed in 70% ethanol, 5% acetic acid, and 4% formaldehyde for 18 h. The samples were subsequently critical-point dried, sputter coated with gold in an ion sputter (E-100, Japan), and observed with a scanning electron microscope (Hitachi S-3000N, Japan).

For TEM assays, the samples were fixed in 2.5% (w/v) paraformaldehyde and 0.25% glutaraldehyde in a 0.2 N sodium phosphate buffer for 2–4 h at 4 °C (pH 7.0), after which they were post-fixed in 1% OsO_4_ in phosphate-buffered saline (PBS; pH 7.4). After being dehydrated in ethanol, the samples were embedded in acrylic resin. Ultrathin sections (50–70 nm) were double stained with 2% (w/v) uranyl acetate and 2.6% (w/v) lead citrate aqueous solution, after which the sections were examined with a transmission electron microscope at 200 kV (Tecnai G2 20 Twin, FEI, The Netherlands).

### RNA extraction and qRT–PCR

Total RNA was extracted with 1 ml Trizol reagent (Invitrogen) according to the manufacturer’s instructions. After isopropanol precipitation, the RNA was resuspended in 30 μl RNase-free water and treated with RNase-free DNase I (New England Biolabs). For quantitative reverse transcription–PCR (qRT-PCR), first-strand cDNA was reverse transcribed using random primers (primers are listed in Supplementary Table S2). Ubiquitin served as a control for gene expression.

### Analysis of RNA editing

For RNA editing analysis in the WT and the *Ospgl1* line, total RNAs were isolated from young leaves using Trizol reagent as previously described (Hu *et al.*, 2012). RNA was treated with RNase-free DNase I (New England Biolabs) and confirmed by PCR. Then, the RNAs were reverse transcribed with random primers and the high-fidelity reverse transcriptase SuperScript III (Invitrogen). Primers were designed to cover all 491 mitochondrial editing sites and 21 chloroplast editing sites (Supplementary Table S2). The RT–PCR products were sequenced directly.

### Subcellular localization of OsPGL1

For transient expression in rice protoplasts, the full-length cDNA of *OsPGL1* was cloned into the HBT-sGFP vector driven by the cauliflower mosaic virus (CaMV) 35S promoter to produce a construct encoding the 35S:OsPGL1:sGFP fusion protein. Protoplast preparation and transformation procedures were as previously described (Yu *et al.*, 2014). MitoTracker Red (Invitrogen) was used as a mitochondrial-specific dye.

### RNA electrophoresis mobility shift assays

The corresponding cDNA fragment of *OsPGL1* was amplified with specific primers (Supplementary Table S2) and cloned into a pGEX-6p-1 vector to create a construct encoding a glutathione S-transferase (GST)-OsPGL1 fusion protein. Two RNA probes (probe 1 and probe 2) and a negative control probe (probe C) containing the target editing site were synthesized and labeled with biotin at the 3ʹ end by GenScript (Nanjing, China). RNA electrophoresis mobility shift assays (REMSAs) were performed as previously described (Hu *et al.*, 2012). Briefly, the recombinant protein was incubated with an RNA probe in a 20 µl reaction mixture that included 10 µl of 2× binding buffer [100 mM Na phosphate (pH 7.5), 10 units of RNasin, 0.1 mg ml^–1^ bovine serum albumin (BSA), 10 mM dithiothreitol, 2.5 mg ml^–1^ heparin, and 300 mM NaCl]. The mixture was incubated at 25 °C for 30 min followed by separation in 5% native PAGE in 0.5× TBE buffer; afterwards, the reactants were transferred to a nylon membrane (Roche). For the competitive REMSAs, gradually increasing concentrations of unlabeled probe were added to the reaction mixture in accordance with the procedure described above.

### Complementation of *Ospgl1* mutants

For complementation of *Ospgl1* mutants, a full-length (1815 bp) cDNA fragment was constructed into a pCAMBIA-2300 vector driven by the CaMV 35S promoter, after which the construct was transformed into an *Ospgl1-1* mutant background by the *Agrobacterium*-mediated method. Independent transgenic lines were obtained and subsequently planted in Wuhan, China.

### Yeast two-hybrid assays

The full-length cDNAs of *OsPGL1* (*Os12g06650*), *WSP1* (*Os04g51280*), *OsMORF1* (*Os11g11020*), *OsMORF2* (*Os06g02600*), *OsMORF3* (*Os03g38490*), *OsMORF8* (*Os09g33480*), and *OsMORF9* (*Os08g04450*) were cloned into the pGBKT7 and pGADT7 vectors. The constructs were subsequently co-transformed in pairs into yeast (strain AH109) in accordance with previously described methods (Hu *et al.*, 2012).

### GST pull-down assays

The purified recombinant proteins (GST tag, Trx-His tag, GST-OsPGL1, WSP1-His, Trx-OsMORF2-His, Trx-OsMORF8-His, and Trx-OsMORF9-His) were dialyzed against PBS (137 mM NaCl, 2.7 mM KCl, 10 mM Na_2_HPO_4_, 2 mM KH_2_PO_4_) for 24 h and quantified using the bicinchoninic acid method. The recombinant GST-OsPGL1 protein was incubated with glutathione sepharose for 1 h on ice and then washed five times with five volumes of PBS. Trx-His, WSP1-His, and Trx-OsMORFs-His proteins were subsequently added to detect any interaction. The bound proteins were washed five times with five volumes of PBS, which were eluted with glutathione reductase and separated by 10% SDS-PAGE. The resulting products were transferred on to a polyvinylidene fluoride (PVDF) membrane (Bio-Rad) and investigated with antibodies to GST and His.

### Bimolecular fluorescence complementation assays

For the bimolecular fluorescence complementation (BiFC) analyses, the full-length cDNA of *OsPGL1* without the stop codon was fused to the C-terminus fragment of yellow fluorescent protein (YFP) in a pUC-SPYCE (C-terminus) vector. Similarly, *OsMORF2*, *OsMORF8*, and *OsMORF9* were fused to the N-terminus fragment of YFP in a pUC-SPYNE (N-terminus) vector. The two vectors were co-transformed in pairs into rice protoplasts and observed by bright-field and fluorescence microscopy using a Leica microscope (DM4000 B, Germany) (Hu *et al.*, 2012).

### Co-immunoprecipitation analysis

The total proteins from transgenic plants fused with FLAG (UBI:OsPGL1-FLAG) and GFP (35S:OsMORFs-GFP) were extracted with extraction buffer (100 mM Tris-HCl, 200 mM NaCl, 5 mM EGTA, 5 mM EDTA, 10 mM dithiothreitol, 0.6% TritonX-100, 1 mM phenylmethylsulfonyl fluoride; pH 8.0). Afterwards, the extracted proteins and 2 µg of anti-GFP antibodies were incubated together overnight at 4 °C; 100 µl of protein A-Sepharose beads was then added, after which the mixture was incubated for an additional 3–4 h. The immunoprecipitates were washed five times with co-immunoprecipitation buffer (150 mM NaCl, 20 mM Tris-HCl, 1 mM EDTA, 0.2% NP-40, 1 mM phenylmethylsulfonyl fluoride; pH 7.4) and loaded into 6× SDS loading buffer by denaturing for 10 min. The proteins were separated via 10% SDS-PAGE and then were detected by immunoblotting with anti-FLAG and anti-GFP antibodies.

### Immunoblot analysis

Total proteins were extracted from young leaves and quantified with a bicinchoninic acid protein assay kit (Thermo Scientific). A 10 μg aliquot of total proteins was separated by SDS-PAGE and then transferred on to a PVDF membrane (Bio-Rad), after which the membrane and primary antibodies to NdhD (Beijing Protein Innovation, China), PsaA, PsbA, PetA, AtpA, Lhca2, and Lhcb2 (Agrisera) were incubated together. Actin served as a reference antibody. Detection was carried out by using ECL western blotting detection reagents (Bio-Rad).

### Blue-native PAGE

The equivalent of 500 µg of total mitochondrial proteins from WT and *Ospgl1-1* mutant plants were treated and loaded on to a blue-native PAGE system as described previously (Liu *et al.*, 2012). The gel was first stained with Coomassie brilliant blue. For immunoblotting, the gel was then transferred to a PVDF membrane, after which antibodies to Cytc1 were used to detect the accumulation of complex III.

### Accession numbers

Sequence data from this paper can be found in the GenBank database under the following accession numbers: OsPGL1 (XP_015618645.1), WSP1 (XP_015636466), NdhD (NP_039444), CcmFc (YP_002000589), OsMORF1 (XP_015615260), OsMORF2 (XP_015643218), OsMORF3 (XP_015628181), OsMORF8 (XP_015612485), and OsMORF9 (XP_015650748).

## Results

### Phenotypic characterization of *Ospgl1* mutants

**Fig. 1.**
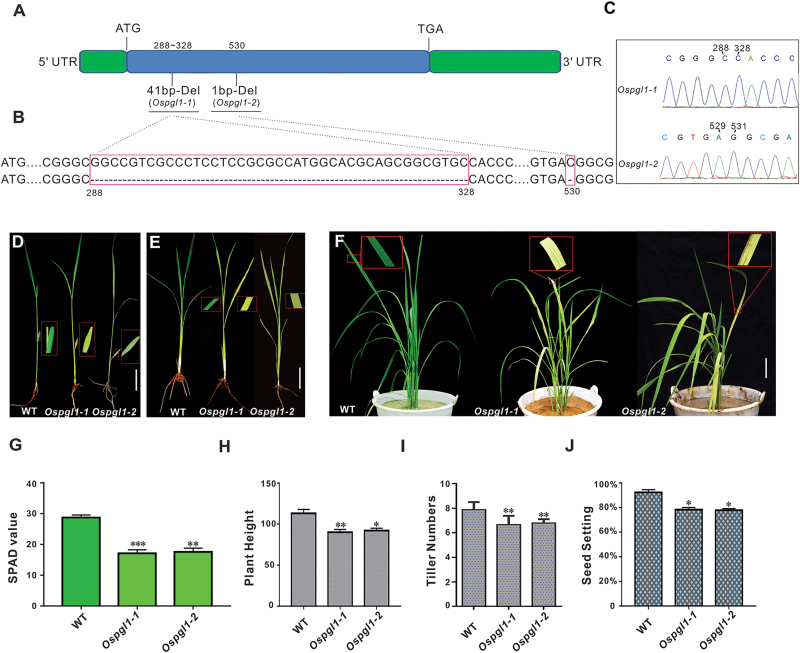
Mutant and phenotypic characterization of *Ospgl1*. (A) Schematic drawing of the intronless gene *OsPGL1* indicating the position of the deletions in the *Ospgl1* mutants. (B) Alignment of WT and mutant DNA sequences around the target sites; the deletion of 41 bp in *Ospgl1-1* and 1 bp in *Ospgl1-2* are highlighted with boxes. (C) Sequencing results of *Ospgl1-1* and *Ospgl1-2* heterozygotes. (D, E) Comparison of leaves from the two *Ospgl1* mutants and a WT plant at (D) the two-leaf stage and (E) the three-leaf stage; the insets show a magnified view. Bar=2cm. (F) Comparison of leaves from the two *Ospgl1* mutants and a WT plant at the tillering stage, the insets show a magnified view. Bar=5cm. (G) Comparison of the chlorophyll content in the two *Ospgl1* mutants and WT plants. Bars represent the mean ±SD of three independent biological replicates. SPAD value is the relative value of chlorophyll content in plant leaves measured by a chlorophyll meter (SPAD-502plus, China). (H–J) Comparison of (H) the plant height, (I) the tiller number, and (J) of the seed setting of the two *Ospgl1* mutants and WT plants. Error bars for (G–J) represent the mean ±SD of approximately 100 plants. Asterisks indicate statistically significant differences compared with WT (*P<0.05, **P<0.01, ***P<0.001; Student’s *t*-test).

We used a CRISPR system to generate several PPR mutants under the background of *Oryza sativa.* L. *japonica* ZH11 and obtained two independent transgenic *Os12g06650* knockout lines. Additional genomic DNA sequencing revealed a 41 bp deletion from position 288 to 328 and a 1 bp deletion at position 530 in this *PPR* gene (Fig. 1A–C), truncating the *Os12g06650* open reading frame and causing two different frameshift mutations at two different target sites. Both lines exhibited pale green leaves at all vegetative stages (Fig. 1D–F); thus, we named this gene *Pale Green Leaves 1* (*OsPGL1*) and defined the two mutant lines as *Ospgl1-1* and *Ospgl1-2*. The chlorophyll content was drastically reduced in the *Ospgl1* lines (Fig. 1G), and this phenotype was more pronounced when they were grown in paddy fields (Supplementary Fig. S1). Despite the occurrence of pale green leaves at all vegetative stages, plant height, number of tillers, and seed setting were not significantly impaired (Fig. 1H–J). To confirm the phenotype of the *Ospgl1* knockout mutants, we also generated transgenic RNAi lines. The results showed that the suppression of *OsPGL1* expression replicated the knockout mutant phenotype (Fig. S2).

### Defective cytological morphology and altered expression of genes related to chloroplast development in *Ospgl1* mutants

**Fig. 2.**
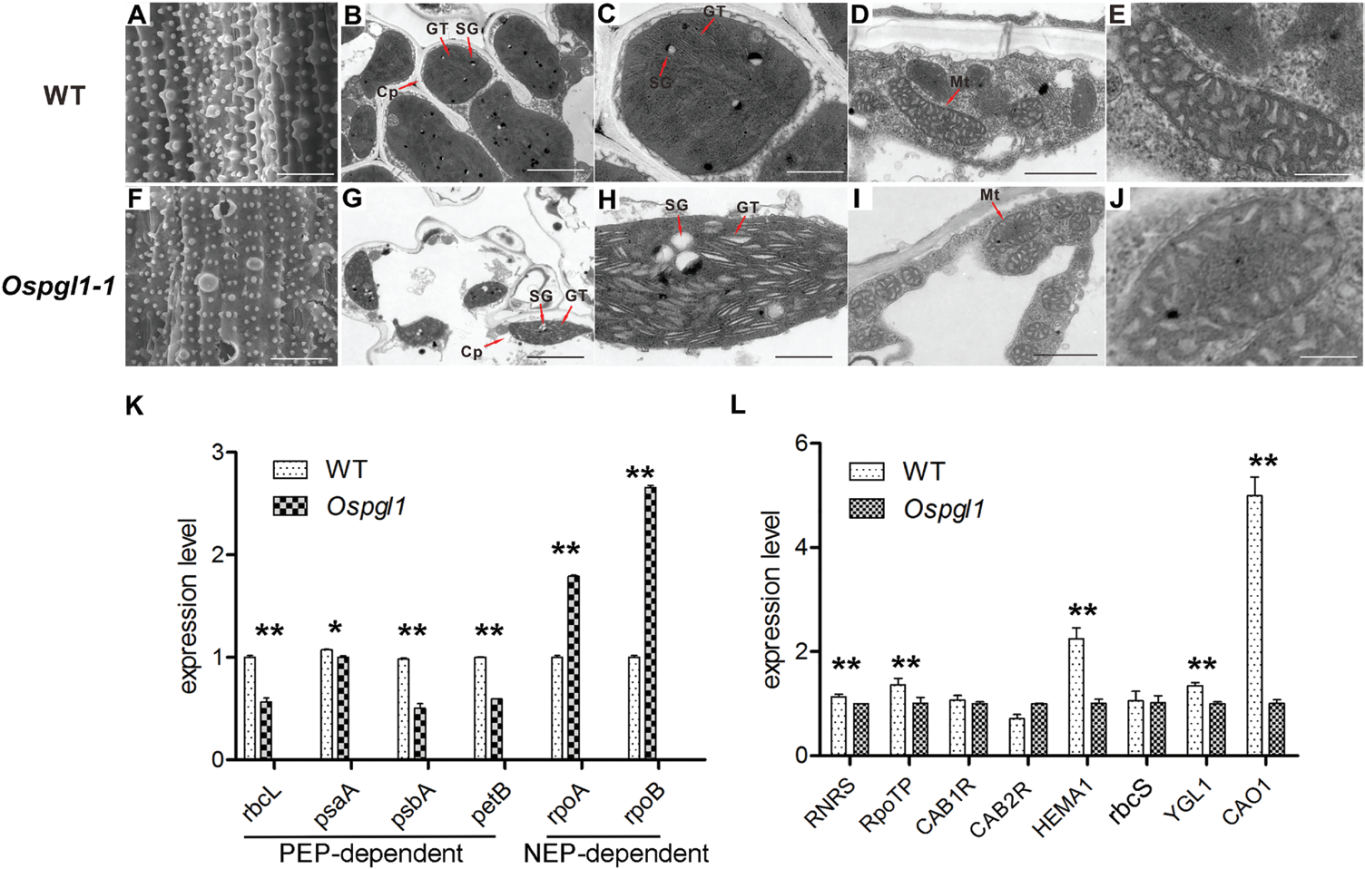
Microstructure observation of leaves at the tillering stage and expression analysis of chloroplast development-related genes in WT and *Ospgl1-1* mutant plants. (A) SEM image of WT leaf. Bar=20 µm. (B) TEM image of WT chloroplasts. Bar=2 µm. (C) A higher magnification image of WT chloroplast from (B). Bar=0.3 µm. (D) TEM image of WT mitochondria. Bar=0.5 µm. (E) A higher magnification image of WT mitochondrion from (D). Bar=0.15 µm. (F) SEM image of mutant leaf. The image shows a defective leaf surface morphology with cracked, broken holes in the pale parts of *Ospgl1-1*. Bar=20 µm. (G) TEM image of chloroplasts in the pale parts of *Ospgl1-1* mutant leaf. The image shows non-compact starch grains and a lack of well-structured grana thylakoid. Bar=1µm. (H) A higher magnification image of chloroplast in the pale parts of *Ospgl1-1* mutant leaf from (G). Bar=0.3 µm. (I) TEM image of mitochondria in *Ospgl1-1* leaf. Bar=0.3 µm. (J) A higher magnification image of *Ospgl1-1* mitochondrion from (I). Bar=0.15 µm. Cp, chloroplast; GT, grana thylakoid; Mt, mitochondrion; SG, starch grain. (K) Real-time RT–PCR examination of PEP-dependent genes (*rbcL*, *psaA*, *psbA*, and *petB*) and NEP-dependent genes (*rpoA* and *rpoB*) in WT and *Ospgl1* plants at the five-leaf stage. (L) Real-time RT–PCR examination of chloroplast development-related and photosynthesis-related genes in WT and *Ospgl1* at the five-leaf stage. Data in (K, L) represent the mean ±SD of three independent biological replicates. *P<0.05, **P<0.01 (Student’s *t*-test).

To determine the morphologic details of the leaves, we performed SEM and TEM examinations. The SEM results showed that the leaves of WT plants were complete and intact (Fig. 2A), while some cracked holes were distributed among the leaves of *Ospgl1-1* (Fig. 2F); these cracked holes may be broken veins caused by defective chloroplasts and reduced chlorophyll content. Furthermore, the TEM assays revealed that starch granular stacks in the WT plants were well balanced (Fig. 2B), while the starch granular stacks in the *Ospgl1-1* plants were reduced and not compact (Fig. 2G). The well-organized grana thylakoids in *Ospgl1-1* were not dense and exhibited more hollow structures compared with WT (Fig. 2C, H). Although the *Ospgl1-1* chloroplasts were impaired, the ultrastructures of the mitochondria were indistinguishable between WT and *Ospgl1-1* (Fig. 2D, E, I, and J). These observations revealed that the *Ospgl1* mutation primarily affects the development and morphology of chloroplasts rather than those of mitochondria.

The development of chloroplasts is related to the coordinated expression of both chloroplastic and nuclear genes. Therefore, we first examined the transcript levels of the chloroplast-encoded genes at the five-leaf stage of *Ospgl1* and WT plants. Most of these genes are mediated by two types of RNA polymerase: plastid-encoded polymerase (PEP) and nuclear-encoded polymerase (NEP). The results showed that the expression levels of PEP-dependent genes (*rbcL*, *psaA*, *psbA*, and *petB*) decreased in *Ospgl1*, which is consistent with their corresponding protein levels (reported below and in Fig. 7); however, NEP-dependent genes, such as RNA polymerase genes *rpoA* and *rpoB*, were up-regulated in *Ospgl1* (Fig. 2K). These results implied that retrograde signals from chloroplasts might up-regulate NEP-dependent gene transcription to compensate for chloroplast development.

In addition to investigating the expression of chloroplast-encoded genes, we investigated the expression of nuclear-encoded genes related to chloroplast development and photosynthesis in *Ospgl1* and WT plants, These genes included *RNRS* (encoding the small subunit of ribonucleoside diphosphate reductase), *RpoTp* (encoding NEP core subunits), *CAB1R* and *CAB2R* (light-harvesting chlorophyll *a*/*b*-binding proteins of PSII), *HEMA1* (encoding a glutamyl-tRNA reductase), *rbcS* (encoding a Rubisco small subunit), *YGL1* (encoding a chlorophyll synthetase), and *CAO1* (encoding chlorophyll *a* oxygenase 1). Our qRT–PCR analysis revealed that, compared with that in the WT plants, the expression level of these genes was lower in the *Ospgl1* mutant plants (Fig. 2L). Taken together, these data indicated that *OsPGL1* plays an important role in regulating chloroplast development and photosynthesis.

### *OsPGL1* encodes a DYW motif-containing protein

*OsPGL1* encodes a putative PPR protein that consists of 604 amino acids and no introns. Motif prediction analysis by Pfam (http://pfam.xfam.org/) revealed that OsPGL1 contains 10 PPR motifs (four S motifs, three P motifs, and three L motifs in a staggered arrangement) (Fig. S3A, B). The C-terminal region from residues 392 to 604 shows a consensus sequence of E, E+ and DYW domains (Fig. S4). These data indicated that OsPGL1 belongs to the typical DYW type of PLS subfamily. Alignment of *OsPGL1* with its orthologs in various plant species showed 75%, 75%, 46%, 51%, 51%, and 47% similarity with *Zea mays* (GRMZM2G001466), *Sorghum bicolor* (Sb08g003980), *Arabidopsis thaliana* (AT4G15720; REME2), *Theobroma cacao* (XP_017974392), *Glycine max* (XP_003529581), and *Brassica napus* (BnaC07g33170D), respectively (Fig. S4). REME2 is involved in RNA editing of *rps3*-1534 and *rps4*-175 in mitochondria (Bentolila *et al.*, 2013*a*). The high similarity among OsPGL1, GRMZM2G001466, and Sb08g003980 implied that the function of this *PPR* gene might be conserved in monocots. We analyzed the conservation of amino acids 1ʹ and 6 of each motif on the basis of the PPR–RNA recognition model; this conservation is essential for RNA recognition. The results showed that these candidate orthologs could be divided into two subgroups, monocots and dicots, which suggested functional conservation (Fig. S5). These results also implied a distinction in function between OsPGL1 and AtREME2.

### Expression pattern and subcellular localization analysis of OsPGL1

**Fig. 3.**
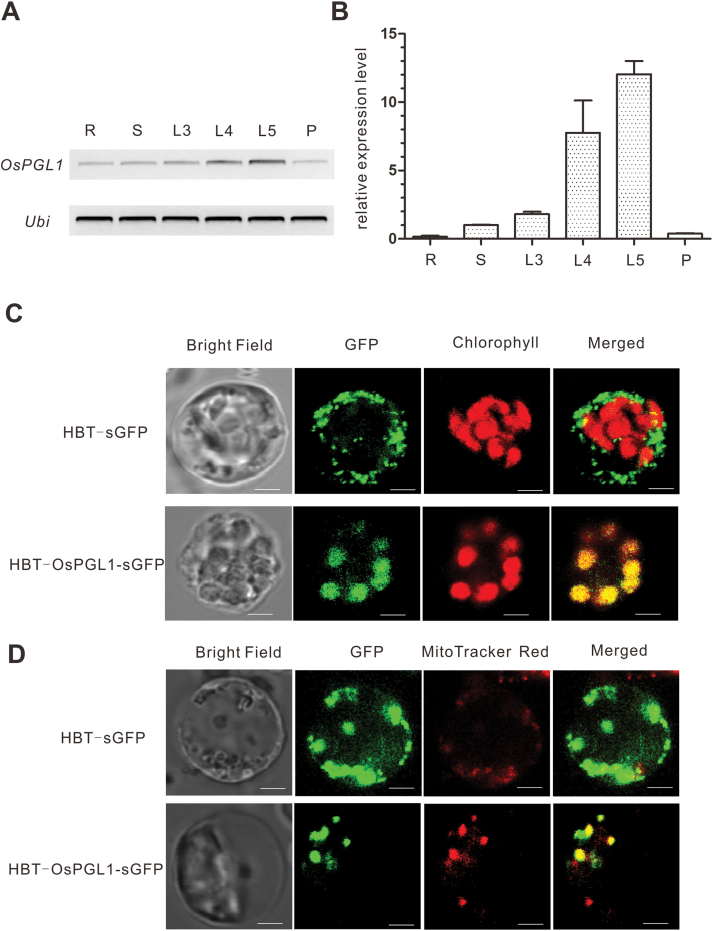
Expression and subcellular localization analysis of *OsPGL1*. (A) RT–PCR examination of *OsPGL1* in different tissues of WT plants. L3, three-leaf stage; L4, four-leaf stage; L5, five-leaf stage; P, panicle; R, Root; S, Stem; Ubi, Ubiquitin (which served as a control gene). (B) Real-time qRT–PCR examination of *OsPGL1* in different tissues of WT plants. Error bars represent the SD. (C) Transient expression of 35S:sGFP (upper panel) and 35S:OsPGL1-sGFP (lower panel) in rice protoplasts. Bar=5 μm. (D) Transient expression of 35S:sGFP (upper panel) and 35S:OsPGL1-sGFP (lower panel) in rice protoplasts. MitoTracker Red was used to label mitochondria. Bar=5 μm.

To gain insight into the expression pattern of *OsPGL1* in WT plants, we performed RT–PCR and qRT–PCR on various tissues. The RT–PCR results showed that *OsPGL1* was constitutively expressed in both vegetative and reproductive tissues, including roots, stems, leaves, and panicles (Fig. 3A). Moreover, the qRT–PCR data also showed that *OsPGL1* was expressed in the roots, stems, and panicles but that its transcript preferentially accumulated in fresh leaves (Fig. 3B). These data are consistent with the results of the leaf phenotypes as well as the effects on plant height and seed setting.

Many reports have confirmed that most PPR proteins are targeted to either plastids or mitochondria. The results of a bioinformatic analysis by TargetP (http://www.cbs.dtu.dk/services/TargetP/) indicated that *OsPGL1* is predicted to localize in the chloroplast and the mitochondrion, albeit with a low degree of confidence. To confirm the subcellular localization of OsPGL1, its full-length cDNA was fused with green fluorescent protein (sGFP) driven by the CaMV 35S promoter, after which the constructs were transiently expressed in rice protoplasts. The results showed that the GFP signals overlapped with the red autofluorescent signals of chlorophyll (Fig. 3C). Interestingly, some GFP spots did not overlap with the signals from the chloroplasts; these spots appear to correspond to mitochondria. MitoTracker Red was subsequently used to label the mitochondria. Signals from the mitochondria also overlapped with those of the OsPGL1-GFP constructs (Fig. 3D). Therefore, these findings indicate that OsPGL1 is a novel rice PPR protein that is dually targeted to chloroplasts and mitochondria.

### C-to-U RNA editing of *ndhD*-878 and *ccmFc*-543 sites are lost in *Ospgl1* mutants and can be restored in transgenic complementation lines

**Fig. 4.**
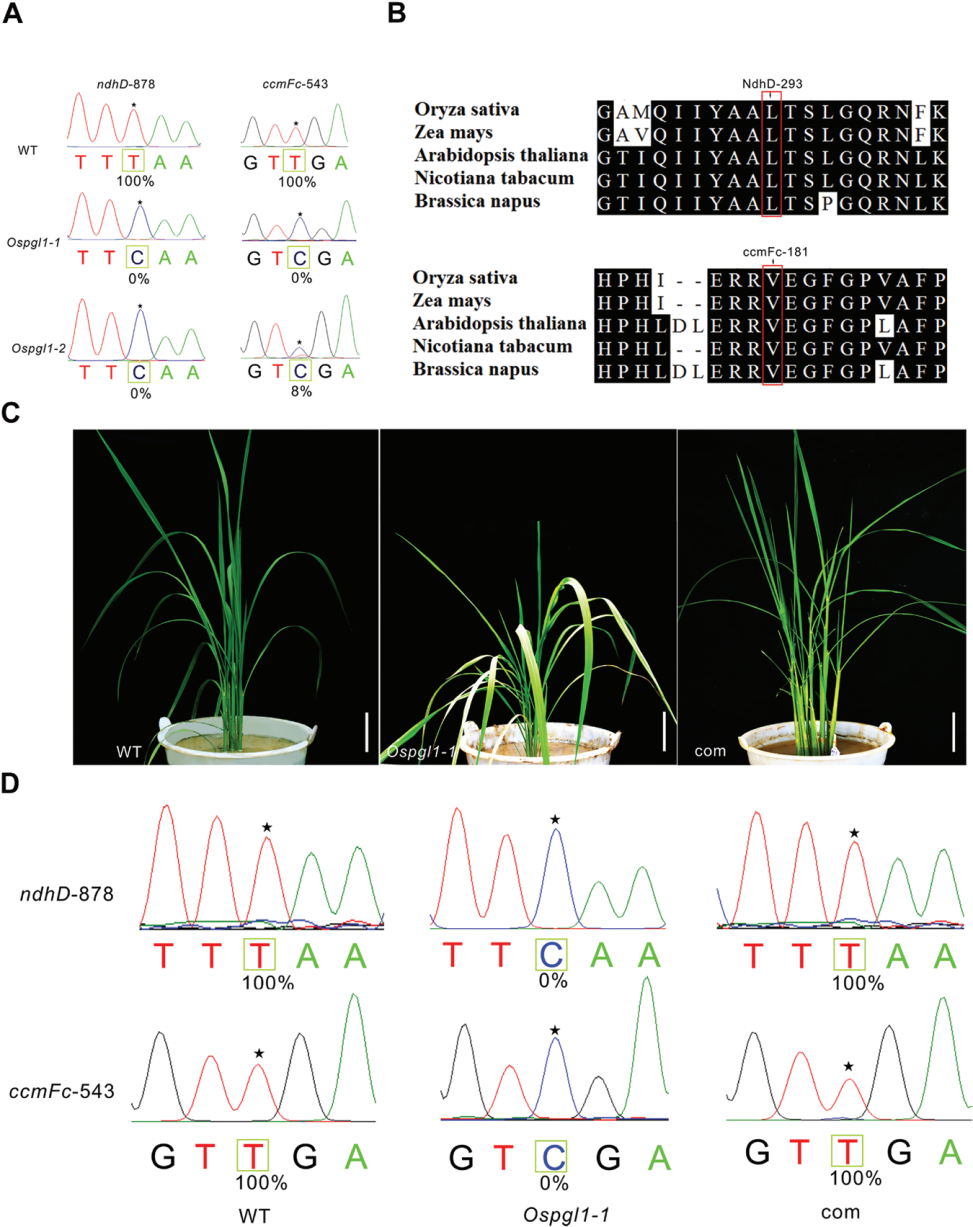
Organelle RNA editing and complementation analysis of the *Ospgl1-1* mutant. (A) RNA editing analysis of the *ndhD*-878 and *ccmFc*-543 sites from WT and *Ospgl1-1* leaves. Asterisks indicate the editing sites, and the editing efficiency is presented under each target site. (B) Alignment of the orthologous NdhD and CcmFc amino acid sequences in five different species around the corresponding affected residues. The converted amino acid is indicated. (C) Phenotypes of WT, *Ospgl1-1*, and complemented T_0_ (com) plants at the tillering stage. Bars=5cm. (D) Comparison of RNA editing efficiency of *ndhD*-878 and *ccmFc*-543 among WT, *Ospgl1-1*, and complemented T_0_ (com) plants; asterisks indicate the editing sites.

PPR proteins are involved in RNA editing at one or several editing sites, especially within transcripts of the DYW subfamily (Sun *et al.*, 2016). Considering the dual localization of OsPGL1, we checked via RT–PCR all 491 editing sites in the mitochondria and 21 editing sites in the chloroplasts. The sequencing results revealed that, with the exception of a value of 8% for *ccmFc*-543 in *Ospgl1-2*, the C-to-U editing efficiency of *ndhD*-878 and *ccmFc*-543 decreased to zero in both mutants, while *ndhD*-878 and *ccmFc*-543 were completely edited in WT (Fig. 4A). The loss of editing at the *ndhD*-878 site failed to convert the codon from UCA to UUA, which resulted in an amino acid substitution of leucine to serine. The loss of editing at the *ccmFc*-543 site failed to convert the codon from GUC to GUU; however, owing to the degeneracy of the codon, the change in nucleotide at that position did not change the amino acid, which is consistent with the lack of observed effects in mitochondria in *Ospgl1* (Fig. 2I, J).

To investigate the evolutionary conservation of the alteration from serine to leucine at position 293 of the NdhD protein sequence and valine at position 181 of the CcmFc protein sequence, the chloroplast NdhD orthologs and mitochondrial CcmFc orthologs from five representative plant species (*Oryza sativa*, *Zea mays*, *Arabidopsis thaliana*, *Nicotiana tabacum*, and *Brassica napus*) were analyzed. The results showed that these two residues are extremely conserved among the five tested species, in both monocots and dicots, implying that the leucine of NdhD and the valine of CcmFc are important for plant development (Fig. 4B).

To verify whether the pale green leaf phenotype and the defective RNA editing observed resulted from the dysfunction of *OsPGL1*, we performed a transgenic complementation assay for the mutant lines. The full-length coding sequence of *OsPGL1* driven by the CaMV 35S promoter was inserted into a pCAMBIA-2300 vector, after which the construct was transformed into *Ospgl1-1* mutants by the *Agrobacterium*-mediated method. All 12 independent transgenic lines completely rescued the mutant pale green leaf phenotype (Fig. 4C). Furthermore, we checked the RNA editing efficiency of *ndhD*-878 and *ccmFc*-543 in all 12 independent transgenic complementation lines; the results showed that 35S:OsPGL1 completely restored the RNA editing efficiency to 100%, the same as the efficiency in WT plants (Fig. 4D). These data indicate that the pale green leaf phenotype was indeed caused by the loss of function of *OsPGL1*.

### OsPGL1 can modularly recognize and bind to both *ndhD* and *ccmFc* transcripts

**Fig. 5.**
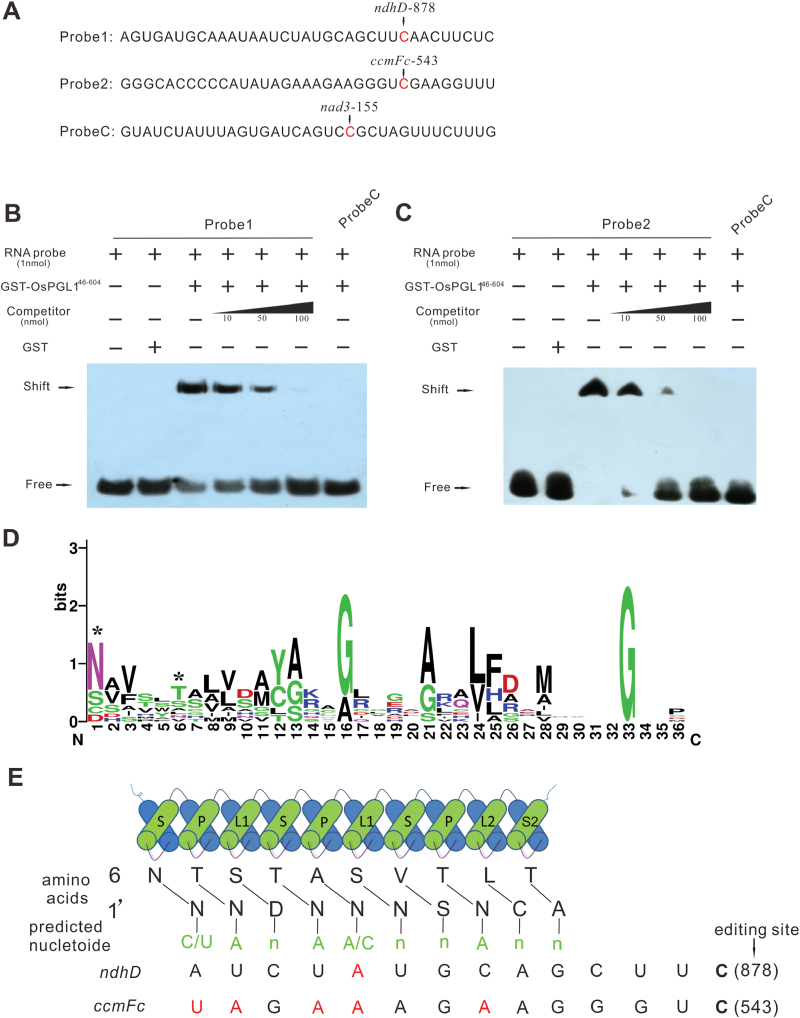
OsPGL1 exhibits specific RNA-binding activities and binds its target RNAs via a modular mechanism. (A) Schematic sequences of RNA probes. Edited sites are indicated. Probe C was used as a negative control. (B) REMSAs of GST-OsPGL1^46-604^ and GST tags, each with RNA probe 1. Unlabeled probe 1 was used as a competitor at a range of concentrations for competitive REMSAs. GST tags and probe C were used as negative controls. (C) REMSAs of GST-OsPGL1^46-604^ and GST tags, each with RNA probe 2. Unlabeled probe 2 was used as a competitor at a range of concentrations for competitive REMSAs. GST tags and probe C were used as negative controls. (D) Sequence logo for PPR motifs in OsPGL1; the two positions that contribute to RNA binding specificity are indicated with asterisks. Sequence logos were constructed by using Web-Logo. (E) Alignment of amino acid residues at positions 6 and 1ʹ in each neighboring PPR motif of OsPGL1 with the putative cis-elements surrounding the editing sites. Nucleotides matching the PPR–RNA recognition combination are indicated with shaded text.

To test the RNA-binding activity of OsPGL1, we expressed recombinant OsPGL1 for use in REMSAs. Ten PPR motifs from residues 46 to 604 were fused with a GST tag for expression in *Escherichia coli*. The recombinant protein (GST-OsPGL1^46-604^) was analyzed by western blot using anti-GST antibodies to confirm its high purity (Supplementary Fig. S6). The recombinant protein was subsequently dialyzed to remove RNAse contamination for REMSAs and further quantified. The *ndhD* and *ccmFc* probes, which included 35 nucleotides surrounding the target editing sites, were prepared and designated as probe 1 and probe 2. Another probe, probe C (*nad3*-155), was used as a negative control; this probe is a specific target of another rice PPR protein (to be reported separately) (Fig. 5A). GST-OsPGL1^46-604^ and biotin-labeled RNA probes were incubated together, and GST tags and the same RNA probes were also incubated together. Both protein–RNA complexes were detected as shifted bands that migrated more slowly than the free RNA probe in the native gel; however, no retarded band was observed when the RNA probe and the GST tag were incubated together (Fig. 5B, C). In addition, as a negative control, no retarded band was observed when probe C and GST-OsPGL1^46-604^ were incubated together. We then performed a competition assay using increasing concentrations of unlabeled RNA probe and a constant concentration of labeled RNA probe of the same sequence; the results showed that the binding intensity of the band decreased as the concentration of unlabeled competitors increased (Fig. 5B, C). These results verified that OsPGL1 binds to both *ndhD* and *ccmFc* transcripts directly via PPR motifs.

PPR proteins bind target RNA via a modular recognition mechanism (Barkan *et al.*, 2012; Yagi *et al.*, 2013). To evaluate the conservation of the P, L, and S motifs of OsPGL1, the sequence of each motif was analyzed. The alignment of these 10 motifs revealed that the threonine, alanine, and serine residues at position 6 as well as the asparagine and aspartate residues at position 1ʹ are conserved polar residues (Fig. 5D). To evaluate the degree of matching of the OsPGL1 protein binding to its two target transcripts, we performed a computational prediction. Alignment of the target sites of OsPGL1 showed comparatively high matches of PPR–RNA recognition on the basis of these two editing sites (Fig. 5E). The alignment results also suggested that the combinations between PPR motifs and their target nucleotides might be flexible.

### OsPGL1 interacts with three OsMORF proteins rather than WSP1

**Fig. 6.**
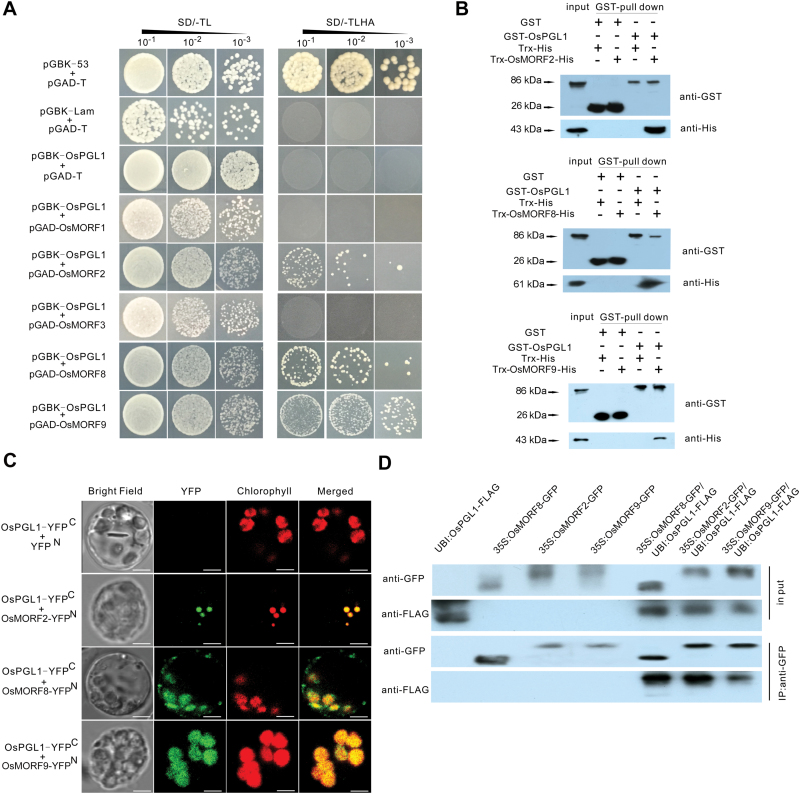
OsPGL1 interacts directly with OsMORF2/8/9 proteins. (A) Yeast two-hybrid assay. pGAD and the GAL4 activation domain were used as a prey vector; pGBK and the GAL4 DNA-binding domain were used as a bait vector. SD/-TL and SD/-TLHA indicate SD/-Trp-Leu and SD/-Trp-Leu-His-Ade dropout plates, respectively. pGBK-53 and pGBK-Lam were used as positive and negative controls, respectively. Co-transformation of pGAD-T and pGBK-OsPGL1 was used for detection of self-activation. (B) GST pull-down assay to detect the interactions between OsPGL1 and OsMORF2/8/9 proteins. GST and Trx-His tag protein were used as controls; the eluates were immunoblotted with anti-GST and anti-His antibodies, respectively. (C) BiFC assay showing that OsPGL1-YFP^C^ interacts with OsMORF2-YFP^N^, OsMORF8-YFP^N^, and OsMORF9-YFP^N^ to produce YFP fluorescence in chloroplasts. Bar=5 µm. (D) Co-immunoprecipitation assay detection with anti-FLAG and anti-GFP antibodies.

A recent study showed that WSP1 is also involved in RNA editing of *ndhD*-878 (Zhang *et al.*, 2017), which is characterized by having a high sequence similarity to OsMORF2 protein. MORF proteins are another group of RNA editing factors in plant organelles. Although the function of these proteins is unknown, interactions between MORFs and several PPR-type RNA editing factors have been demonstrated (Takenaka *et al.*, 2012). To determine whether WSP1 can interact with OsPGL1, we employed yeast two-hybrid and pull-down assays. No interaction was observed in either of these tests (Supplementary Fig. S9). Therefore, we further investigated the interaction between OsPGL1 and other OsMORF proteins in pairs. We first performed yeast two-hybrid assays; the results showed that OsPGL1 can interact with three OsMORFs in yeast: OsMORF2 (Os06g02600), OsMORF8 (Os09g33480), and OsMORF9 (Os08g04450). However, no interaction was observed between OsPGL1 and the other two OsMORFs, OsMORF1 (Os11g11020) and OsMORF3 (Os03g38490), or the negative control (Fig. 6A). Interestingly, the interaction between OsPGL1 and OsMORF9 was much stronger than that with the other OsMORFs. Moreover, interactions between OsPGL1 and OsMORF2/8/9 were also observed after we switched the bait and prey in a yeast two-hybrid system (Supplementary Fig. S7). The results indicated that, compared with the other pairs, the pair of OsPGL1 and OsMORF9 exhibited the strongest interaction.

Next, we performed GST pull-down assays to validate the interactions *in vitro*. The three OsMORF proteins were fused with a His tag. The recombinant proteins were further verified by western blots (Supplementary Fig. S8) and subjected to pull-down assays in pairs. Trx-OsMORF2-His, Trx-OsMORF8-His, and Trx-OsMORF9-His were each pulled down by GST-OsPGL1, which demonstrated that OsPGL1 can interact with these three OsMORF proteins directly (Fig. 6B).

To test the interactions *in vivo*, we performed BiFC assays using rice protoplasts. OsPGL1 and each of three OsMORFs were fused to the C-terminus and N-terminus of YFP, respectively. The results showed that co-expression of OsPGL1-YFP^C^ and OsMORF2-YFP^N^/OsMORF8-YFP^N^/OsMORF9-YFP^N^ exhibited strong signals that overlapped with those from chlorophyll, while the negative combination of OsPGL1-YFP^C^ and YFP^N^ produced no detectable fluorescence signal (Fig. 6C). Next, we generated transgenic plants that each harbored UBI:OsPGL1-FLAG, 35S:OsMORF2-GFP, 35S:OsMORF8-GFP, or 35S:OsMORF9-GFP. The crude protein extracts of these plants and anti-GFP antibodies were incubated together for co-immunoprecipitation assays. The results confirmed the interactions between OsPGL1 and OsMORFs (Fig. 6D). Taken together, these results demonstrated that OsPGL1 interacts with OsMORF2 (Os06g02600), OsMORF8 (Os09g33480), and OsMORF9 (Os08g04450) both *in vitro* and *in vivo*, without interacting with WSP1.

### *Ospgl1* exhibits defective photosynthetic complexes

**Fig. 7.**
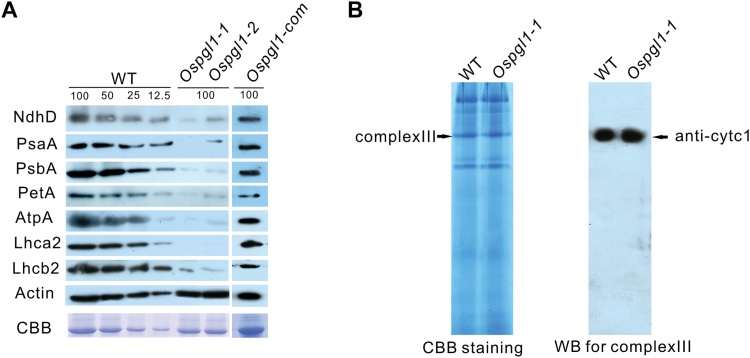
Immunoblot analysis of the subunits of photosynthetic complexes and respiratory complex III. (A) Immunoblot analysis of the subunits of photosynthetic complexes in WT plants, *Ospgl1* mutants, and complementation lines at the four-leaf stage. Anti-actin served as a reference antibody, and Coomassie brilliant blue (CBB) staining was used to indicate the loading control. The lanes were loaded with a series of dilutions, as indicated. (B) The levels of the major proteins of mitochondrial respiratory complex III, as analyzed by blue-native PAGE (left) and immunoblotting (right). Cytc1 served as a representative subunit of complex III.

To clarify whether the photosynthetic and respiratory complexes were impaired in *Ospgl1* mutants, we measured the levels of proteins involved in photosynthesis and the electron transport chain. First, we examined NdhD in WT plants, *Ospgl1* mutants, and complementation lines. The results revealed highly reduced accumulations of NdhD in the *Ospgl1* mutants, suggesting that the loss of editing of *ndhD*-878 generated unstable NdhD in chloroplasts (Fig. 7A). The accumulation of the photosystem I (PSI) subunit PsaA, the photosystem II (PSII) subunit PsbA, cytochrome b6f (PetA), the chloroplast ATP synthase subunit AtpA, the light-harvesting complex of PSI (Lhca2), and the light-harvesting complex of PSII (Lhcb2) were also examined. The levels of all of these proteins dramatically decreased in both mutant lines (Fig. 7A). As expected, the levels of NdhD and the other subunits in the photosynthetic complexes were restored in the transgenic complementation line (Fig. 7A).

We then examined the proteins involved in the mitochondrial electron transport chain. Mitochondrial complexes isolated from *Ospgl1-1* and WT calli were separated on blue-native gels. Coomassie blue staining revealed no definitive changes in any of the complexes, implying that *Ospgl1* did not compromise the function of the mitochondria (Fig. 7B). Although the loss of RNA editing of *ccmFc*-543 did not change the amino acid at the position corresponding to the editing site, we also evaluated mitochondrial complex III, since CcmFc is required for complex III maturation. Protein immunoblotting with anti-CytC1 antibodies revealed no differences in the amount of complex III between *Ospgl1-1* and WT (Fig. 7B), which is consistent with the indistinguishable morphological structures of mitochondria in *Ospgl1* and in WT leaves. Taken together, these results indicate that photosynthetic complexes were impaired in the *Ospgl1* mutants but the respiratory complexes were not affected.

## Discussion

### OsPGL1 is a dual-localized PPR protein involved in RNA editing

PPR proteins are trans-acting factors with respect to organellar RNA and act as site-specific RNA-binding proteins (Okuda *et al.*, 2006). Most PPRs are involved in either mitochondrial or chloroplastic RNA editing; few are dually localized. SOAR1 is a cytosol–nucleus dual-localized PPR protein that acts downstream of CHLH/ABAR and upstream of the nuclear abscisic acid-responsive bZIP transcription factor ABI5 (Jiang *et al.*, 2015). PNM1 is dually localized to mitochondria and nuclei in Arabidopsis; this protein is associated only with polysomes, plays a role in translation in mitochondria, and interacts with TCP8 in the nucleus (Hammani *et al.*, 2011). PPR2263 and MEF29 are dually targeted to mitochondria and chloroplasts, and are required for RNA editing in maize and Arabidopsis, respectively (Sosso *et al.*, 2012). Here, we first report a novel PPR protein that is dually localized to mitochondria and chloroplasts in rice. Our data showed that OsPGL1 is also required for RNA editing and that OsPGL1 binds to target RNA directly. Both *ndhD*-878 (chloroplast) and *ccmFc*-543 (mitochondrion) were completely edited in WT plants, despite the editing of *ccmFc*-543 (mitochondrion) being synonymous. The loss of function of OsPGL1 reduced the editing efficiency to zero and resulted in a pale green leaf phenotype. Arabidopsis PPR CRR28 is required for editing at the chloroplast *ndhD*-878 site (Okuda *et al.*, 2009), and the rice ortholog CRR28 is also a DYW-type PPR, although its function is unclear. The dual localization of OsPGL1 suggested that the signal peptide sequence of OsPGL1 could be applied for synchronously translocating foreign proteins to both mitochondria and chloroplasts.

### OsPGL1 recognizes target RNAs and functions in conjunction with OsMORF proteins

The PPR motif consists of two antiparallel α-helices, and its binding specificity depends on the first α-helix (Barkan *et al.*, 2012). The results of bioinformatic and structural analyses have indicated that two positions of amino acids distributed between two adjacent PPR repeats are highly important for target RNA base recognition (Takenaka *et al.*, 2013*a*). The alignment results of OsPGL1 revealed high conservation of these positions in monocots (Supplementary Fig. S5), implying a function of the orthologous genes involved in RNA editing of *ndhD* and *ccmFc* in monocots.

Non-PPR editing factors and PPO1 are both components of the plant RNA editosome, which is required for RNA editing (Sun *et al.*, 2016). In Arabidopsis plastids, both of the plastid-localized members MORF2 and MORF9 are required for RNA editing at most sites (Takenaka *et al.*, 2012). A recent study has shown that the RNA-binding activity of an artificial (PLS)_3_PPR could be sharply increased upon MORF9 binding, suggesting that interactions between PPR and MORF9 could be more important than those of other proteins (Yan *et al.*, 2017). In Arabidopsis, CLB19 interacts with MORF2; PDM2 interacts with MORF2/9, PDM1/SEL1, and PPO1; and ORRM6 interacts with MORF2/8/9 (Zhang *et al.*, 2014; Ramos-Vega *et al.*, 2015; Zhang *et al.*, 2015; Du *et al.*, 2017; Hackett *et al.*, 2017). Several PPR-type RNA editing factors in Arabidopsis have been demonstrated to interact with MORFs. MEF13 interacts with MORF1 and MORF3 and is involved with eight mitochondrial editing sites in Arabidopsis (Glass *et al.*, 2015). MEF10 interacts with MORF8 and is required for the RNA editing of *nad2*-842 in Arabidopsis mitochondria (Härtel *et al.*, 2013). MEF35 interacts with MORF1 and MORF8, and is required for the RNA editing of three sites in Arabidopsis mitochondria (Brehme *et al.*, 2015). All these data indicate that the editosome in plants is complex. Nevertheless, MORF proteins in rice are rarely reported. WSP1, a MORF2-like (Os04g51280) protein, was recently identified in rice; this protein is involved in the RNA editing and splicing of several plastid genes (Zhang *et al.*, 2017). WSP1 is also involved in the RNA editing of *ndhD*-878, suggesting that WSP1 and PGL1 might cooperate together for RNA editing of *ndhD*-878. Therefore, we tested the interaction between WSP1 and OsPGL1. The results of yeast two-hybrid assays and pull-down assays showed that OsPGL1 and WSP1 did not interact together *in vitro* (Supplementary Fig. S9). However, we confirmed that OsPGL1 can interact with OsMORF2 (Os06g02600), OsMORF8 (Os09g33480), and OsMORF9 (Os08g04450) both *in vitro* and *in vivo*. The interaction between OsPGL1 and OsMORF2 was seemingly weaker but significantly stronger than that between OsPGL1 and the other OsMORFs (Fig. 6A, Supplementary Fig. S7), implying that the contributions of different OsMORFs to the RNA-binding activities of OsPGL1 differ. The results of our studies suggest that OsPGL1 performs organellar RNA editing via an editosome coupled with OsMORF proteins. By using our transgenic plants harboring UBI:OsPGL1-FLAG, 35S:OsMORF2-GFP, 35S:OsMORF8-GFP, or 35S:OsMORF9-GFP, we will explore other factors or subunits of the editosome in future studies.

### OsPGL1 plays an important role in rice chloroplast development

Leaves constitute an important plant tissue. In this study, we constructed CRISPR/Cas9 knockout mutants lacking *OsPGL1*. Both *Ospgl1-1* and *Ospgl1-2* mutants exhibited pale green leaves throughout the whole vegetative stage. Further investigation revealed that chloroplast development and photosynthesis were defective at the RNA and protein levels in the mutants. The up-regulation of NEP-dependent genes in the mutants implied that unknown retrograde signals from the chloroplasts were involved in compensatory effects during the development of chloroplasts. Complementation lines could rescue this aberrant phenotype, suggesting that the base deletion mutation in the *OsPGL1* gene was responsible for the pale green leaf phenotype. OsPGL1 regulated chloroplast development by its organellar RNA editing of *ndhD*. The highly conserved leucine at NdhD-293 is important for the structure or function of NdhD. The results of protein immunoblotting showed that the amounts of the photosynthetic complex subunits dramatically decreased in the mutants. To date, there is not enough evidence to prove that the defect of RNA editing in ndhD transcripts and loss of the NDH complex can cause the pale green phenotype; thus, the pale green leaf phenotype of the *Ospgl1* mutant might be caused by the absence of other functions of OsPGL1, which require further study. However, the results suggest that *PPR* genes play a vital role in regulating chloroplast development in plants.

## Supplementary data

Supplementary data are available at *JXB* online.

Fig. S1. Phenotype of the WT and the *Ospgl1-1* mutant in a paddy field.

Fig. S2. Phenotype of the WT and an *Ospgl1* RNAi line.

Fig. S3. Schematic structural sequence of OsPGL1.

Fig. S4. Sequence alignment of *OsPGL1* with its orthologs in various plants.

Fig. S5. Conservation of the amino acid at the 6 and 1ʹ positions of each PPR motif.

Fig. S6. Expression and purification of GST-OsPGL1^46-604^.

Fig. S7. Interaction of OsPGL1 with OsMORF2/OsMORF8/OsMORF9 detected by yeast two-hybrid assays.

Fig. S8. Expression and purification of Trx-OsMORF2-His, Trx-OsMORF8-His, and Trx-OsMORF9-His.

Fig. S9. Interaction of OsPGL1 with WSP1 detected by yeast two-hybrid assays and pull-down assays.

Table S1. Design of target adaptor for CRISPR/Cas9 system.

Table S2. Primers used for qRT–PCR, vector construction, and RNA editing.

Supplementary Materials

## Author contributions

HX, J Hu, and YZ designed the study. HX and QZ contributed to the construction of the *Ospgl1-1* and *Ospgl1-2* mutants and RNAi lines. HX and YX carried out most of the experiments, HX and QZ conducted SEM and TEM, FZ and CN performed expression and purification of recombinant proteins, and J Huang, WL, and LP contributed to field management. HX and J Hu wrote the manuscript with feedback from all authors.
