# Supplementary material for: A rice dual-localized pentatricopeptide repeat protein is involved in organellar RNA editing together with OsMORFs
Source: J Exp Bot. 2018 Mar 17;69(12):2923–36. doi: 10.1093/jxb/ery108 (PMC5972581; doi:10.1093/jxb/ery108)
Supplement: Supplementary Materials [file ery108_suppl_supplementary-figures.pdf]

A Rice Dual-localized Pentatricopeptide Repeat Protein is involved in Organellar RNA Editing together with OsMORFs

Haijun Xiao, Yanghong Xu, Chenzi Ni, Qiannan Zhang, Feiya Zhong, Jishuai Huang, Wei Liu, Leilei Peng, Yingguo Zhu, and Jun Hu *

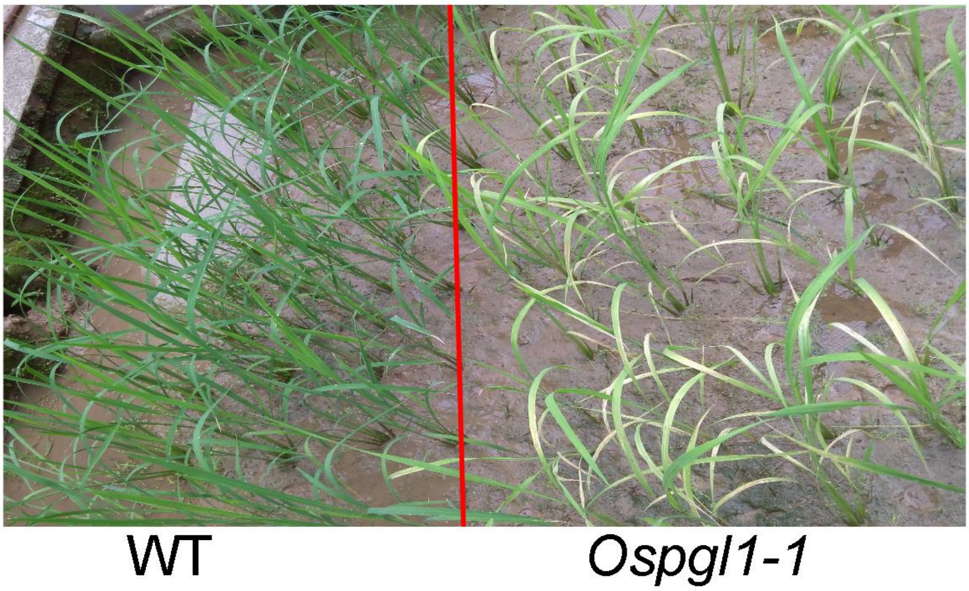

Fig S1. Phenotype of the WT and *Ospgl1-1* mutant in a paddy field.

WT (Left) and *Ospgl1-1* mutant (Right) plants are planted in a paddy field under natural conditions.

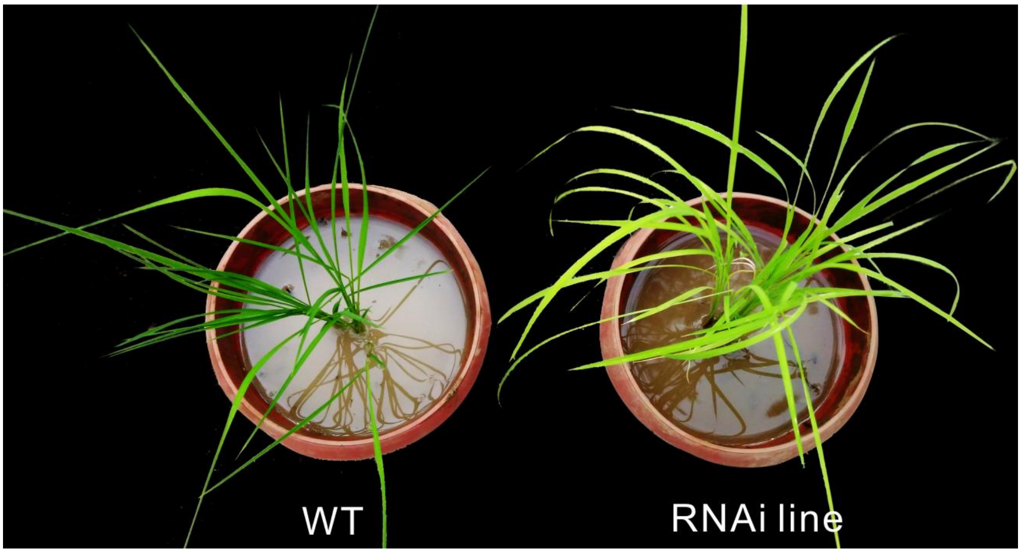

Fig S2. Phenotype of the WT and an *Ospgl1* RNAi line.

A comparison of WT (Left) plant and an *Ospgl1* RNAi line (Right) at the seedling stage.

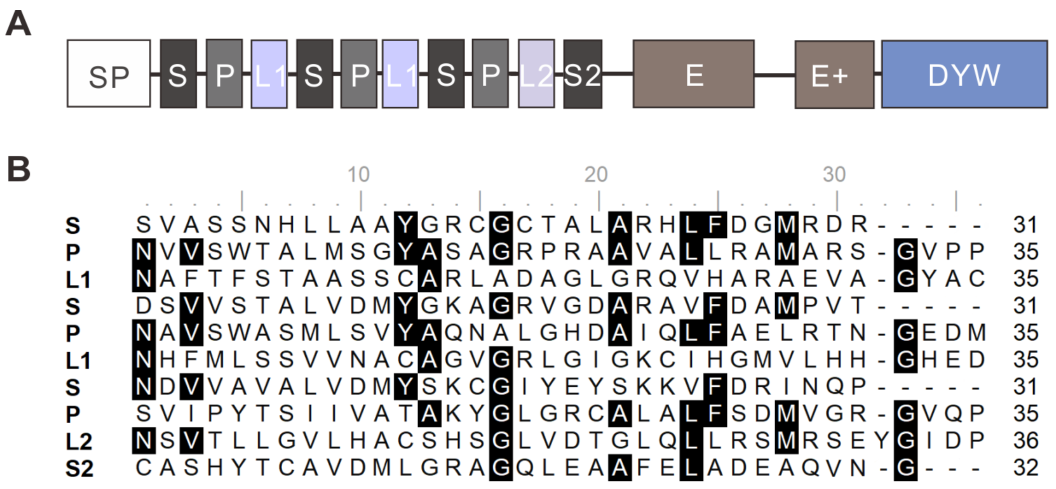

Fig S3. Schematic structural sequence of OsPGL1.

(A) OsPGL1 contains ten tandem PPR motifs (4 s motifs, 3 p motifs and 3 L motifs), and E, E+, DYW domains at the C terminus.

(B) Alignment of the ten PPR motifs of OsPGL1 shows highly conserved amino acids in several positions.

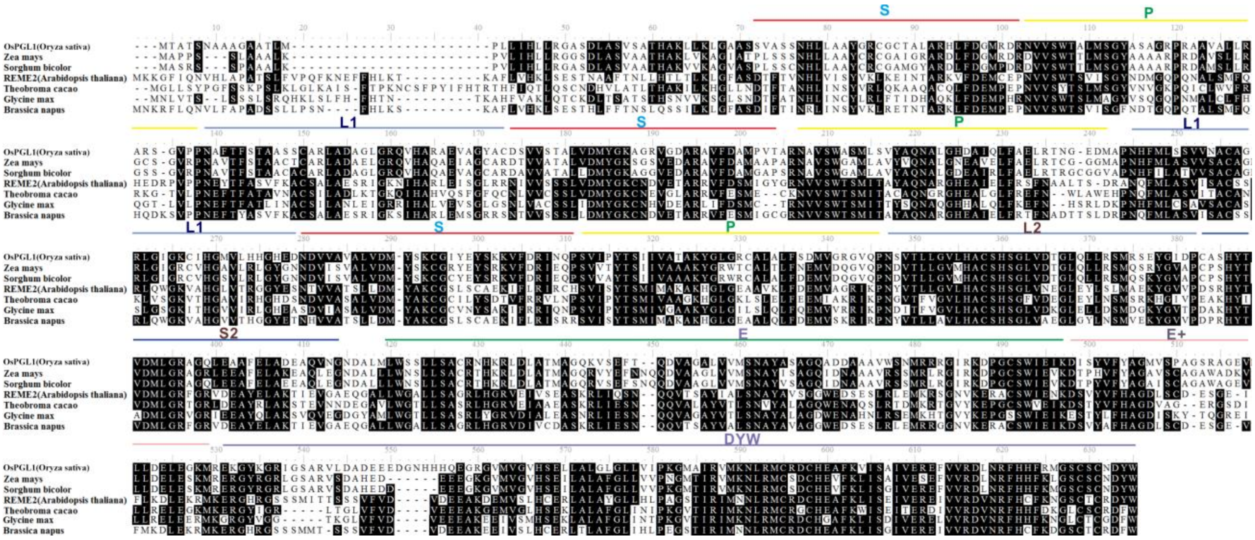

Fig S4. Sequence alignment of *OsPGL1* with its orthologs in various plants.

Alignment of rice *OsPGL1* with its orthologs from *Zea mays*, *Sorghum bicolor*, *Arabidopsis thaliana*, *Theobroma cacao*, *Glycine max* and *Brassica napus*. The 10 PPR motifs and the E, E+, DYW domains are pointed out by the straight lines.

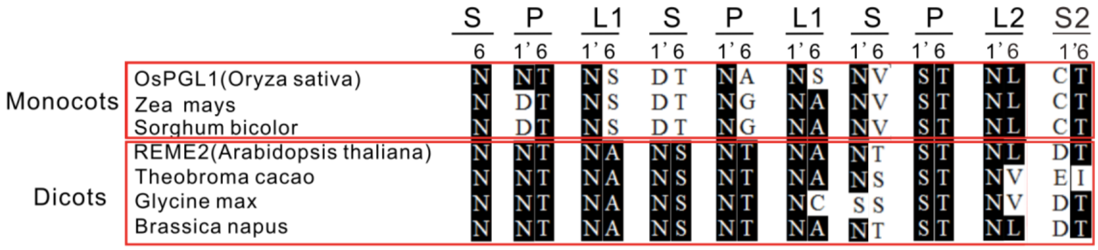

Fig S5. Conservation of the amino acid at 6 and 1' positions of each PPR motif.

Alignment of 6 and 1' amino acids of each motif, which are essential for RNA recognition in *Zea mays*, *Sorghum bicolor*, *Arabidopsis thaliana*, *Theobroma cacao*, *Glycine max* and *Brassica napus*.

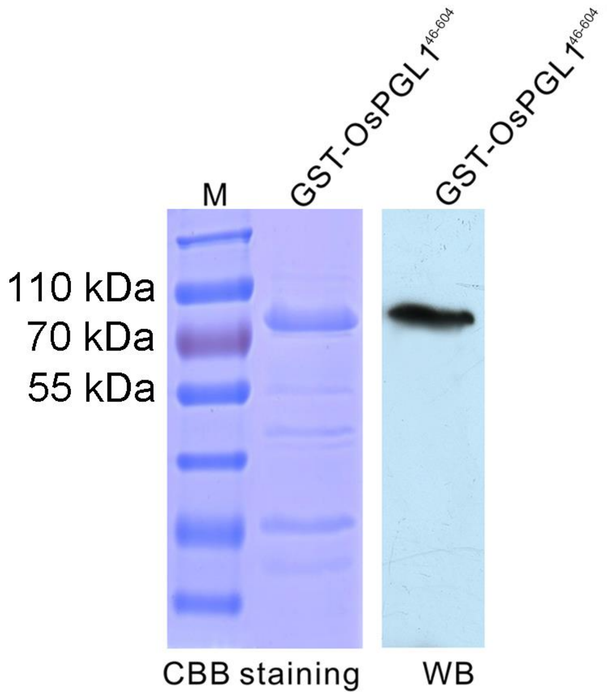

Fig S6. Expression and purification of GST-OsPGL1$^{46\text{-}604}$.

The expression and purification of GST-OsPGL1$^{46\text{-}604}$ (left) and western blot validation with antibody anti-GST (right). Coomassie brilliant blue (CBB), western blot (WB).

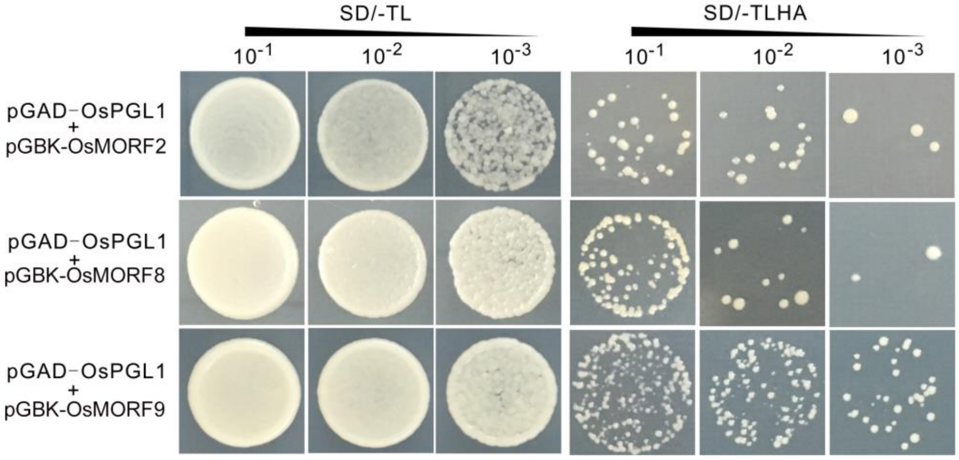

Fig S7. Interaction of OsPGL1 with OsMORF2/OsMORF8/OsMORF9 detected by yeast two-hybrid assays.

Full-length CDS of *OsPGL1* gene was cloned into pGAD (served as prey vector). Full-length CDS of *OsMORF2/8/9* genes were cloned into pGBK (served as bait vector). The constructs were co-transformed into yeast strain AH109. SD/-TL and SD/–TLHA indicate SD/-Trp-Leu and SD/-Trp-Leu-His-Ade dropout plates, respectively. The ability to grow on SD/-TLHA plates indicates an interaction between each two proteins.

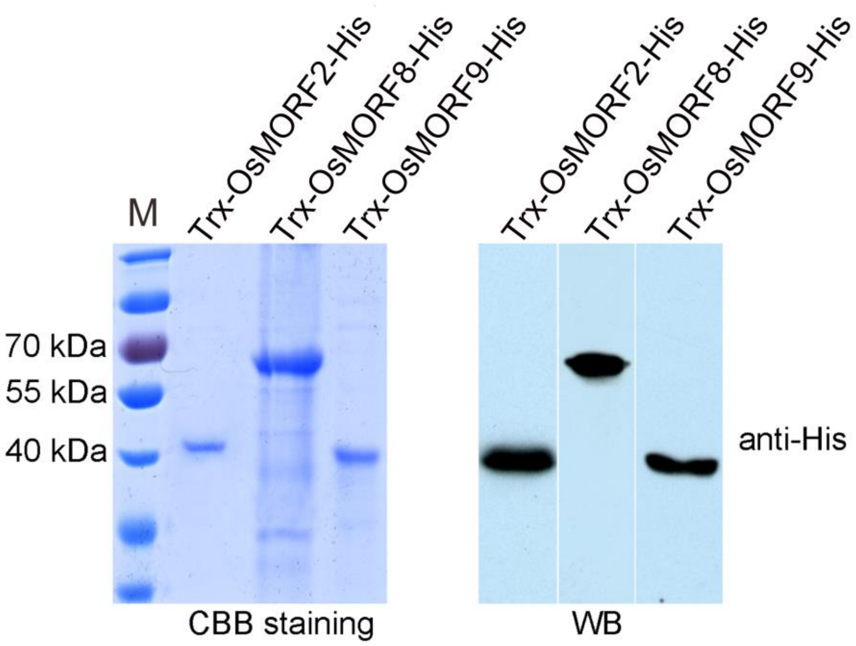

Fig S8. Expression and purification of Trx-OsMORF2-His, Trx-OsMORF8-His and Trx-OsMORF9-His.

The expression and purification of Trx-MORF2-His, Trx-MORF8-His and Trx-MORF9-His (left) and western blot validation with antibody anti-His (right). Coomassie brilliant blue (CBB), western blot (WB).

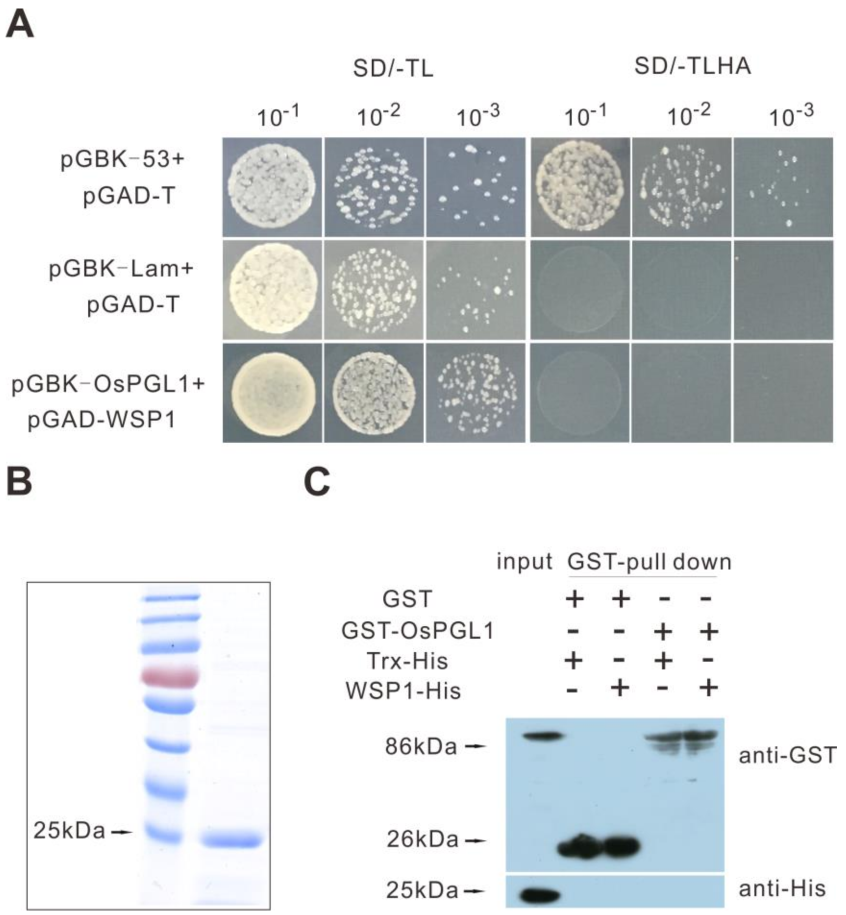

Fig S9. Interaction of OsPGL1 with WSP1 detected by yeast two-hybrid assays and pull-down assays.

(A) Yeast two-hybrid assays: Full-length CDS of *WSP1* gene was cloned into pGAD (served as prey vector). The constructs were co-transformed into yeast strain AH109. SD/-TL and SD/–TLHA indicate SD/-Trp-Leu and SD/-Trp-Leu-His-Ade dropout plates, respectively. The inability to grow on SD/-TLHA plates indicates no interaction between this two proteins.

(B) The expression and purification of recombinant WSP1-His protein detected by Coomassie blue staining.

(C) GST pull-down assays: the interactions between OsPGL1 and WSP1 proteins were detected by GST pull-down assays. GST and Trx-His tag protein were used as controls; the eluates were immunoblotted with anti-GST and anti-His antibodies, respectively.

Table S1. Design of target adaptor for CRISPR/Cas9 system

name	sequence
A target adaptor F1(U3)	GGCAACCGCGTTTCTCGCCGTCA
A target adaptor R1(U3)	AAACTGACGGCGAGAAACGCGGT
A target adaptor F2(U6)	GCCGCTCCGCGCCATGGCACGCAG
A target adaptor R2(U6)	AAACCTGCGTGCCATGGCGCGGAG
OsPGL1-gRT-U3+	5'-ACCGCGTTTCTCGCCGTCAGTTTTAGAGCTAGAAAT-3'
OsPGL1-gRT-U6a+	5'-CTCCGCGCCATGGCACGCAGGTTTTAGAGCTAGAAAT-3'
OsPGL1-OsU3T-	5'-TGACGGCGAGAAACGCGGTTGCCACGGATCATCTGC-3'
OsPGL1-OsU6aT-	5'-CTGCGTGCCATGGCGCGGAGCGGCAGCCAAGCAGCA-3'

The underlined sequences are the enzyme restriction site.

Table S2. Primers used for qRT-PCR, vector construction and RNA editing.

Primers used for mitochondrial RNA editing assay		
**genes**	**Forward sequence (5’-3’)**	**Reverse sequence (5’-3’)**
*Atp*1-1	ATGGAATTCTCACCCAGAGC	TATAGGAACCAGGCTATCCA
*Atp*1-2	TAGAAAGAGCCGCTAAAC	CTAATTAATCTCCTTCGCAG
*atp*6-1	AATTACTCATTTTGATGGAG	CCACTGCCATTAGCACCTTT
*atp*6-2	GAAAATGACTTGTCACTGTG	TTTCGATCACAATCATGTGG
*atp*9	GCAAAGTCAAGTCTCCACGA	CAAAGAGAGATATCTACACC
*ccm*B	ATGAGACGACTCTTTCTTGA	TCAATCTTGTGAACTAATCG
*ccm*C	ATGTCAGTTTCGTTATTACA	CTAGGTTTTTAGTGGTATTC
ccmFc-1	GGTCCAACTACAGAACTTCT	AGTAGTCGTGACCAACAGCCA
ccmFc-2	TGTTGGTCACGACTACTACAAAAAA	TCGATTCCAATGCAACTTAT
ccmFn-1	ATGTCTATAAATGAATTTTC	AACCACGGGAGCGCCCAGCG
ccmFn-2	TTGACGGAGCTCTTGCCATT	CGAGCTTCTTATATGGGATC
ccmFn-3	CAGGACCAGGAACCAATTCG	CTACGGACGGGACGAAATCC
cob-1	ATGACTATAAGGAACCAACG	AGCCAGATGAAGAAGACTGG
cob-2	CATTGGGTGTACATTCTGAG	CTAAGAGACTGATCCGGTGC
*cox*1	AATGCTCTGAGCAGTTTCGG	CTAGCTTTTTGTCTCTTTGA
*cox*2	GTATAGTAGTCTCATTGGCC	TCTTTCAAAGTCACCGCTTC
*cox*3	TATTACCAAGCACCCTCCAC	TCATATACCTCCCCACCAAT
*nad*1-1	AGGCCCGATCATGAGTGAAT	GAAAATGATCTGGTTGGACG
*nad*1-2	CGTCCAACCAGATCATTTTC	TCATATTGGCATACTCTCCC
*nad*1-3	GGGAGAGTATGCCAATATGA	TTAAGGGAGCCATCGAAAGG
*nad*2-1	CCCACTTCGATCAATTAGCC	CGCTATATATTTGACACGGG
*nad*2-2	GCTCTAGCCAAAACGAATCC	TGCAGGGAAACTTGCACGTG
*nad*3	ATGGACAACATTTTTTTTGG	TTACTCCCGATCCGAAGCAC
*nad*4-1	TAGAACATTTCTGTGAATGC	CCCATACCCCTATAATGATG
*nad*4-2	GCCCATATGAATTTGGTGAC	CACTAAGTTACTTACGGATG
*nad*4L	ACGGATCCTATAAAATATTT	TTAACCTTGAATGCAATTTA
*nad*5	GGGAGTCTCTTTGTAGGATA	TAAGAAAACTGCTCACTAAC
*nad*6	ATGATACTTTCAGTTTTGTC	TTAGTAGATCGTGATTTGGT
*nad*7-1	TGACGACTAGGAACGGGCAA	CCACTAATCGTTGTTTCCAG
*nad*7-2	CACAGCAAGCAAAGGATTGG	CTATCTATCTACCTCTCCAAACACA
*nad*9	ATGGATAACCAATCCATTTT	TTATCCGTCGCTACGCTGTT
*orf*25	ATGGGATTGAGTTCAACGGA	CTTTCACTCACTGAAAAGTG
*Orf*B	ATGCCTCAACTTGATAAATT	TTAGATTATGCTTCCTTGCC
*Orf*X	GCCGAAAATGCATTTATCCT	CCACAAAGATAGCAAACTCG
*rpl*16	ATGGAAAAACATCTTGTAAT	TTACGACCACTGAACAAACT

*rps*11	ACGAAAAGAGTAAGAGCGCA	GAAAGAGGGATGATGCCTAC
*rpl*2	ATCCAGGTCAAGGCGCAAAG	TTTCTAAGCTTACGTGCACC
*rpl*5	ATGTTTCCACTCCATTTTCA	GATCGAAACGACTTTCCTGC
*rps*1	ATGTTCTTGGTGGATGCAGG	TCAAGTTCTTGTTTGATCTG
*rps*2	TGAAAAAGACCAATCAAATCAAACT	GGGTTCGTGCACAGATTTAC
*rps*3-1	GGCACGAAAAGGAAATCCAA	TATTAAAAAAGTATTGCATG
*rps*3-2	CTCTTTCCTTTCTTCGGTGC	ATTTCGTACGTTTCGGATATAGCAC
*rps*4-1	ATGCCTGCATTAAGATTTAA	AGTTGTGAGTAAGCGGAACC
*rps*4-2	GCGGAAAACCGAAAAAGAGC	TTATATGTTTTGGCCACGTC
*rps*7	ATGGGGGACTTTGATGGTGA	TTACCACCATCTGAAATGCG
*rps*13	ATGTCATATATCTCAGGAGC	TCATTTCCGAATTAGCTTGC
*rps*19	ATGCCACGACGATCTATATG	TTACTTTTTCCCCTTTCTGC
**Primers used for chloroplast RNA editing assay**		
**genes**	**Forward sequence (5'-3')**	**Reverse sequence (5'-3')**
*atp*A	CCCAGGGGATGTTTTTTATT	TGAAAAAAGCGTCCATTGTC
*ndh*A	ATGATAATAGACAGGGTACAGG	TTATAGTGAAACAAGTTGGGAAG
*ndh*B	ATGATCTGGCATGTACAGAATG	CTAAAAGAGGGTATCCTGAGCA
*ndh*D	ATTTTGGCTTCCTTATTGC	GCCTCTACCCTGTCAACG
*ndh*F	ATATGCATGGGTAATCCCTC	AGTGGCTCCTAAGAAAAGTG
*ndh*G	ATGGATTTACCTGGGCCAAT	TTATTGCCGAGCCATAGTAA
*rpl*2	ACGGCGAAACATTTATACAA	TTACTTACGGCGACGAAGAATA
*rpo*B	ACTAAGCGTGCTATTCTCAA	TTTATGGTCTAATTCCGAGC
*rps*8	ATGGGCAAGGACACTATTG	AACATAAGACTTCTCCCCCA
*rps*14	ATGGCAAAAAAAAAGTTTGATTC	TTACCAACTGGATCTTGTTGCA
*ycf*3	ATGCCTAGATCCCGTATAAATG	TTATTCAAATTCAAAGCGCTTC
**Primers used for RT-PCR and qRT-PCR**		
**genes**	**Forward sequence (5'-3')**	**Reverse sequence (5'-3')**
OsPGL1-RT	TGATGAAGAAGAGGATGGAA	GTGGTGGAACCTGTTGAGAT
OsPGL1-qRT	CAACGCCTTCACCTTCTCTA	CTTGCCGTACATGTCGACGA
rbcL	CTTGGCAGCATTCCGAGTAA	ACAACGGGCTCGATGTGATA
psaA	GCGAGCAAATAAAACACCTTTC	GTACCAGCTTAACGTGGGGG
psbA	CCCTCATTAGCAGATTCGTTTT	ATGATTGTATTCCAGGCAGAC
petB	ATTCAGACCTCGCAACCAGA	GTTGGCCTCGGTCATTATGT
rpoA	CCATTCCCACAAGCAAAAAT	TCTTACCGCCTTCCGTAGAA
rpoB	AAGAATTCCTGACTATTGCATGGGAA	GTTAGAGCGCCGATGGGTAAC
RNRS	ACGTGCTCGCCTTCTTCG	CAGCGCATGGCCCAGT
RpoTP	GTTGAACGTGAGTTCAAAGTCC	GGGTCCCTAA CTTCACTTGGG
CAB1R	AGATGGGTTTAGTGCGACGAG	TTTGGGATCGAGGGAGTATTT

CAB2R	TGTTCTCCATGTTCGGCTTCT	GCTACGGTCCCCACTTCACT
HEMA1	CGCTATTTCTGATGCTATGGGT	TCTTGGGTGATGATTGTTTGG
rbsC	TCCGCTGAGTTTTGGCTATTT	GGACTTGAGCCCTGGAAGG
YGL1	AACCTTACCGTCCTATTCCTT	CCATACATCTAACAGAGCACC
CAO1	GTTGAACGTGAGTTCAAAGTCC	GGGTCCCTAA CTTCACTTGGG
Ubi	ACCACTTCGACCGCCACTACT	ACGCCTAAGCCTGCTGGTT
**Primers used for Subcellular localization**		
**Primer name**	**Forward sequence (5'-3')**	**Reverse sequence (5'-3')**
OsPGL1-Loc	GGATCCATGACGGCGACATCCAACGCC	GGATCCCAGCATCGAGGCCCAGGAA
**Primers used for prokaryotic expression （pull-down assay）**		
**Primer name**	**Forward sequence (5'-3')**	**Reverse sequence (5'-3')**
OsPGL1-Exp1	GAATTCGACAGCGTCGTTTCCACTGCGCT	CGGCCGCCAGTAGTCATTGCAAGAACA
MORF2-Exp	GGATCCATGGCCGCCGCCGCCGCCGCCGCCG	GGATCCCCGTTGGTTCTCCCTCCTCCGGGC
MORF8-Exp	GGATCCATGGCGTCGGCGTCGCGCT	GGATCCCTGGTAATTCCTCCCTTGTC
MORF9-Exp	GGATCCATGGCTTCCTTCCCGACCACC	GGATCCTGAAGAAGAGGCTGACTCAG
WSP1- Exp	TAAGAAGGAGATATACCATGGGCATGGCCACCGCCGCAGCAGCA	TGGTGGTGGTGGTGCTCGAGCCGCTGGTACGCCTGATTCT
**Primers used for Yeast Two-Hybrid Analysis**		
**Primer name**	**Forward sequence (5'-3')**	**Reverse sequence (5'-3')**
OsPGL1-Y2H	CATATGATGACGGCGACATCCAACGCCGCCG	GAATTCTCACCAGTAGTCATTGCAAGAACAT
MORF1-Y2H	CTCAGAGGAGGACCTGCATATGATGGCCCTCGCGCTGCGACTCC	ACGGATCCCCGGGAATTCTTATTGCCTTCCCCGCCAGC
MORF2-Y2H	CATATGATGGCCGCCGCCGCCGCCGCCGCCG	GAATTCTCACCGTTGGTTCTCCCTCCTCCG
MORF3-Y2H	CTCAGAGGAGGACCTGCATATGATGGCGGCCGGAGCAGCAGCA	ACGGATCCCCGGGAATTCCTACTGCTGCAGATTTGAAG
MORF8-Y2H	CATATGATGGCGTCGGCGTCGCGCTTCCTCC	GAATTCCTACTGGTAATTCCTCCCTTGTCCG
MORF9-Y2H	CATATGATGGCTTCCTTCCCGACCACCGCCG	GAATTCTCATGAAGAAGAGGCTGACTCAG
WSP1- Y2H	CATATGATGGCCACCGCCGCAGCAGCA	GAATTCTCACCGCTGGTACGCCTGATTCT
**Primers used for BiFC assays**		

**Primer name**	**Forward sequence (5’-3’)**	**Reverse sequence (5’-3’)**
OsPGL1-Bi	ATCGATCCAGTAGTCATTGCAAGAACATGAC	ATCGATCCAGTAGTCATTGCAAGAACATGAC
MORF2- Bi	GGATCCATGGCCGCCGCCGCCGCCGCCGCCG	GGATCCCCGTTGGTTCTCCCTCCTCCGGGC
MORF8- Bi	GGATCCATGGCGTCGGCGTCGCGCTTCCTCC	GGATCCCTGGTAATTCCTCCCTTGTCCGT
MORF9- Bi	GGATCCATGGCTTCCTTCCCGACCACCGC	GGATCCTGAAGAAGAGGCTGACTCAGGCT
**Primers used for transgenic expression (Co-IP assays)**		
**Primer name**	**Forward sequence (5’-3’)**	**Reverse sequence (5’-3’)**
OsPGL1-OE	AGATCTATGACGGCGACATCCAACGCCGCCG	AGATCTCCAGTAGTCATTGCAAGAACATGAC
MORF2- OE	GGATCCATGGCCGCCGCCGCCGCCGCCGCCG	GGATCCCCGTTGGTTCTCCCTCCTCCGGGC
MORF8- OE	GGATCCATGGCGTCGGCGTCGCGCTTCCTCC	GGATCCCTGGTAATTCCTCCCTTGTCCGT
MORF9- OE	GGATCCATGGCTTCCTTCCCGACCACCGC	GGATCCTGAAGAAGAGGCTGACTCAGGCT
**Primers used for transgenic complementation analysis**		
**Primer name**	**Forward sequence (5’-3’)**	**Reverse sequence (5’-3’)**
OsPGL1-com	GGTACCATGACGGCGACATCCAACGCCGCCG	CTGCAGTCACCAGTAGTCATTGCAAGAAC
**Primers used for antisense transgenic RNAi**		
**Primer name**	**Forward sequence (5’-3’)**	**Reverse sequence (5’-3’)**
OsPGL1-RNAi	AGATCTATGACGGCGACATCCAACGCCG	CCATGGTCACCAGTAGTCATTGCAAGA

The underlined sequences are the attB recombination sites in the Gateway System (extended line), enzyme restriction site (short line) and homologous arm sequence used for recombination reaction (double underline).
